# Targeted disruption of supraspinal motor circuitry reveals a distributed network underlying Restless Legs Syndrome (RLS)-like movements in the rat

**DOI:** 10.1038/s41598-017-10284-3

**Published:** 2017-08-29

**Authors:** Chun-Ni Guo, Wen-Jia Yang, Shi-Qin Zhan, Xi-Fei Yang, Michael C. Chen, Patrick M. Fuller, Jun Lu

**Affiliations:** 1 Department of Neurology, Shanghai First People’s Hospital Affiliated to Shanghai Jiaotong University, Shanghai, China; 2 Shanghai Yueyang Integrated Medicine Hospital, Shanghai, China; 3 Department of Neurology, Xiuwu Hospital, Capital Medical University, Beijing, China; 4 Shenzen Centers for Disease Control and Prevention, Guangdong, China; 5 Department of Neurology and Division of Sleep Medicine, Beth Israel Deaconess Medical Center and Harvard Medical School, CLS 709, 3 Blackfan Circle, Boston, MA 02115 USA

## Abstract

In this study we uncovered, through targeted ablation, a potential role for corticospinal, cerebello-rubro-spinal, and hypothalamic A11 dopaminergic systems in the development of restless legs syndrome (RLS)-like movements during sleep. Targeted lesions in select basal ganglia (BG) structures also revealed a major role for nigrostriatal dopamine, the striatum, and the external globus pallidus (GPe) in regulating RLS-like movements, in particular pallidocortical projections from the GPe to the motor cortex. We further showed that pramipexiole, a dopamine agonist used to treat human RLS, reduced RLS-like movements. Taken together, our data show that BG-cortico-spinal, cerebello-rubro-spinal and A11 descending projections all contribute to the suppression of motor activity during sleep and sleep-wake transitions, and that disruption of these circuit nodes produces RLS-like movements. Taken together with findings from recent genomic studies in humans, our findings provide additional support for the concept that the anatomic and genetic etiological bases of RLS are diverse.

## Introduction

Movement and motor activity is strongly suppressed during NREM and REM sleep as well as during sleep-wake transitions. The appearance of motor activity during sleep is a common pathologic feature of several parasomnias, in particular restless legs syndrome (RLS) and REM sleep behavior disorder (RBD) characterized by disinhibited movements during REM sleep^1^. Although animal and human research has provided a more detailed understanding of the brainstem neural circuitry regulating REM sleep atonia and RBD^2–4^, the neural circuitry underlying RLS remains poorly understood^5^.

Pathologic movements of RLS occur during both NREM sleep and sleep-wake transitions. These movements of the limbs and body are thought to be precipitated by uncomfortable feelings, which are intensified during the late day and earlier night, and result in insomnia and daytime sleepiness^6, 7^. Our previous work in rodents has suggested that dysfunction of pontine and medullary reticulospinal systems that support normal REM sleep atonia may underlie RLS. However, lesions of these structures that are sufficient to disturb motor activity during REM sleep do not alter motor activity during NREM sleep and sleep-wake transition periods^8–11^. Thus supra-pontine structures with direct projections to the spinal cord may play a more critical role in RLS, including the corticospinal tract, rubrospinal tract and hypothalamic A11 dopaminergic cell group. Basal ganglia (BG) dysfunction has also been implicated in RLS^12^. The BG consisting of several interconnected structures that process cortical inputs and regulate cortical activity to influence a myriad of functions, including motor and sleep behaviors^13^. Interestingly, the hypothalamic A11 cell group is the only dopaminergic source to the spinal cord that has been implicated in RLS^14^, although definitive proof that this cell group contributes to RLS is lacking^15, 16^. Canonical models of BG function posit a key role for the thalamus in relaying BG signals to the cortex, yet more recent studies have identified a direct pallidocortical projection from the globus pallidus externa (GPe) to the cortex^17–19^, suggesting that GPe relays the BG signals to regulate cortical activity. Within the BG, the GPe is regulated by the substantia nigra pars compacta (SNc), with dopamine playing a central role in this interaction^13^.

To systematically explore the potential roles and contributions of three supra-pontine descending projections in RLS, we placed discrete lesions within the 1) corticospinal tract and its sources (motor cortex and somatosensory cortex), or 2) the red nucleus (RN) and its afferent cerebellar interposed nucleus (IP) or 3) hypothalamic A11 dopaminergic cell group, and examined the effects of these lesions on motor activity and sleep-wake structure in the rat. In a subset of animals that developed RLS, we administered the dopamine D2/D3 agonist pramipexole, a drug of choice for treating human RLS, to determine the efficacy of this drug in our rat model of RLS. Finally, to investigate the potential roles and contributions of the BG in RLS, we placed lesions within the SNc, striatum, GPe, and pallidocortical neurons and examined the effects of these lesions on motor activity and sleep-wake structure in the rat.

## Results

### Role of forebrain descending projections in RLS

#### Role of corticospinal tract (CST) in RLS

To identify the role of the CST in the development of RLS-like movements, we placed lesions in three different regions, using three separate groups: corticospinal tract at the C1 level (N = 5), motor cortex (M2) (N = 6) and somatosensory cortex (SS1) (N = 6). In addition, we placed lesions into the hippocampus to serve as anatomical control, i.e., both M2 and SS1, but not the adjacent hippocampus, provide corticospinal projections^20^.

**Figure 1:**
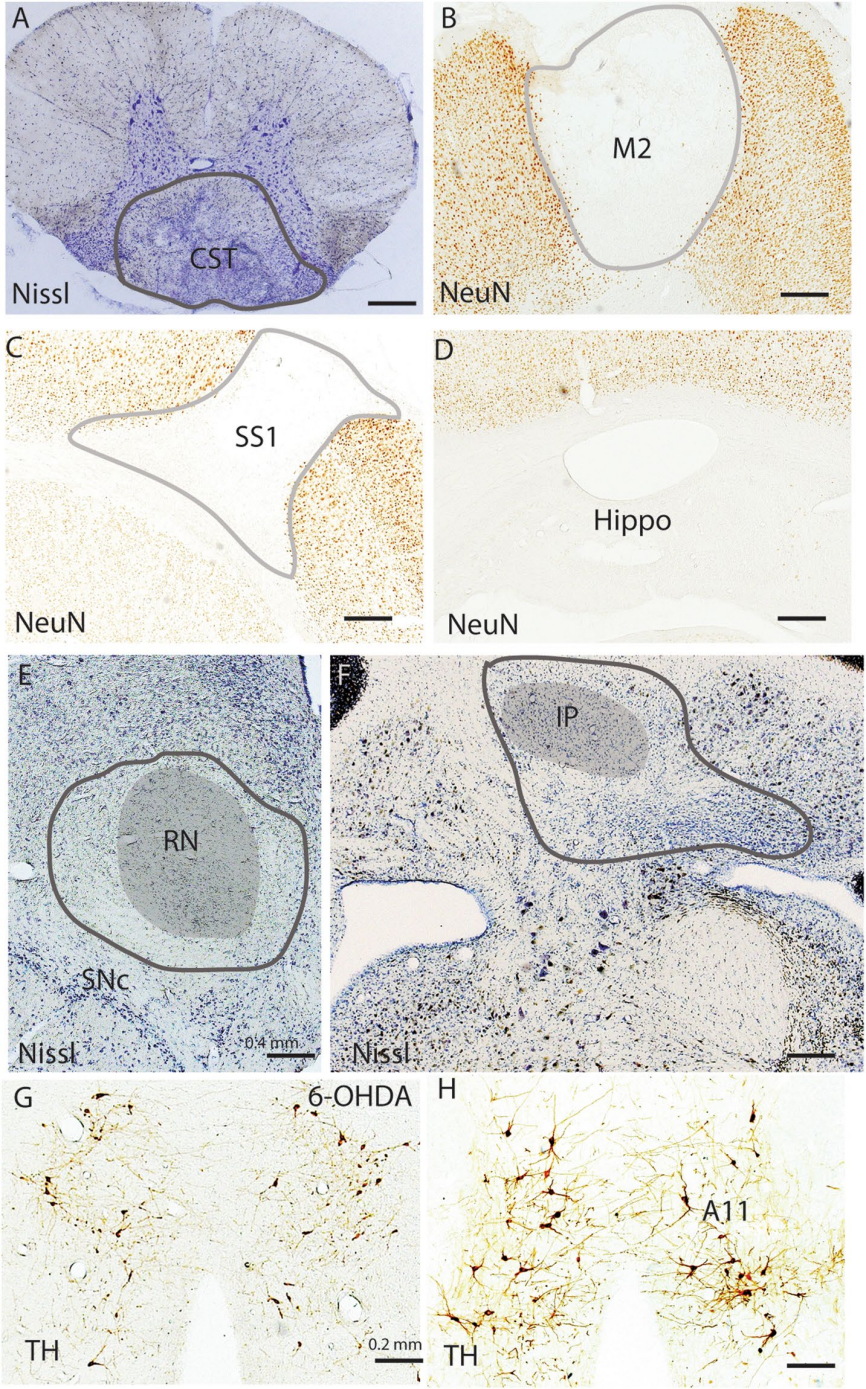
Histological tissues of typical lesions in CST, M2, SS1 and hippocampus, RN, IP, and A11. CST lesion is made by a knife cut at C1 level, which selectively eliminates CST to spinal cord below the C1 level (**A**). Lesions in M2 (**B**), SS1(**C**), hippocampal (**D**), RN (**E**) and IP (**F**) are created by ibotenic acid. Because we selectively destroy A11 descending projections by 6-OHDA injections into the dorsolateral spinal cord at C1 level (**G**) and A11 has diverse projections, the remained neurons are likely those projecting to extra-spinal cord sites. Lesion areas are outlined. Lesions in M2, SS1 and hippocampus are obvious as the lesion areas are white (lack of NeuN staining) while lesions in CST, RN and IP are distinctly different in loss of Nissl labeled neurons and lesions are bigger than RN and IP. Loss of TH-ir neurons in A11 is determined by counting TH positive cells in 6-OHDA injection in the spinal cord (**G**) and control (**H**).

To maximally denervate cortical inputs to the entire spinal cord, we transected the dorsal portion of the spinal cord at the C1 level where the corticospinal tract passes through (Fig. 1). Two weeks after the coronal transection, we recorded EEG/EMG/video for 24 hours. For controls, we injected saline into the third ventricle in 8 rats.

**Figure 2:**
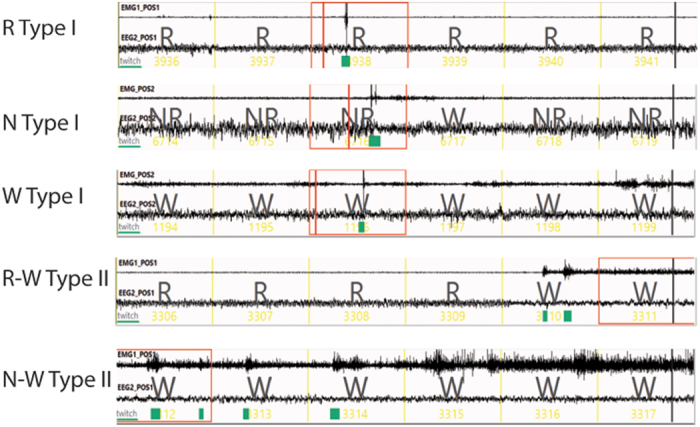
Examples of RLS-like movements and distribution of RLS-like movements across 24 hours. Muscle activity (EMG) is shown in the upper thin line and cortical activity (EEG) is shown in thick line below in 60 seconds segment (10 seconds/unit). NR = NREM sleep, W = wake and R = REM sleep. Type I movements were characterized by singular, abrupt and violent jerking movements, whereas type II movements were characterized by clustered (2–5) movements and slow twitching. Type I movements occured only during REM sleep in normal rats while type I movements during wake and NREM sleep and type II movements were almost never observed in intact (control) rats. Although intact rats occasionally exhibited an EMG trace with type II movements, the movements were always very small. Type I and type II movements were combined in our quantification to define RLS-like movements.

**Figure 3:**
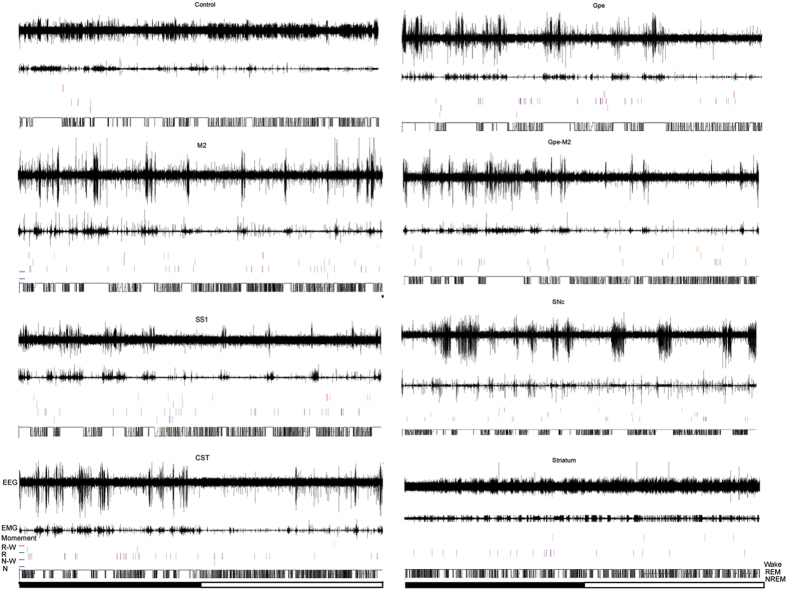
Distributions of RLS-like movements in control and lesion (M2, SS1, CST, GPe, GPe-M2, SNc, and striatum) animals across a day (black bar = light-off period; white bar = light-on period). 24 hour EEG/EMG trace, sleep-wake stages and timing of RLS-like movements indicate that far more RLS-like movements occur in sleep and sleep-wake transitions in lesion groups than control. Overall, RLS-like movements mostly occur during sleep-wake (N-W, R-W) transitions and were vigorous during the second half of the night.

**Figure 4:**
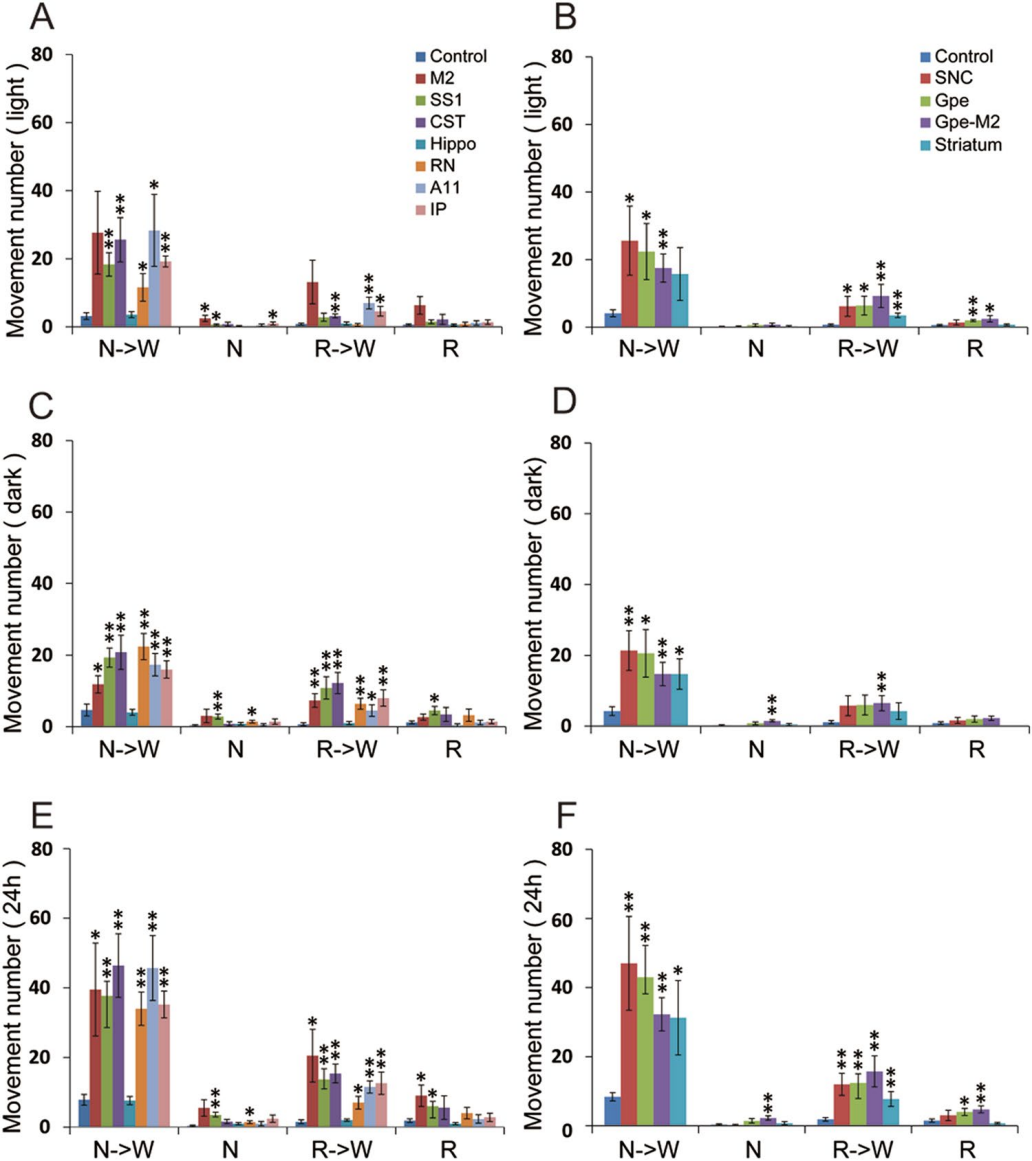
Day-night amounts of RLS-like movements of lesion of corticospinal systems, A11 and BG. Damage to the corticospinal system (CST, M2 and SS1), cerebellorubrospinal system (IP and RN) and A11 descending projections, but not hippocampus, induced significant RLS-like movements. RLS-like movements were predominately induced during sleep-wake transition (N-W and R-W) and overall RLS-like movements were more vigorous during the night than the daytime. Similarly, damage to BG structures (SNc, striatum, GPe, GPe-M2) resulted in RLS-like movements. During NREM sleep and REM sleep, these lesions tended to increase RLS-like movements; however, only M2, SS1, RN and GPe-M2 lesioned groups showed statistical significance. *p < 0.05, **p < 0.01.

**Figure 5:**
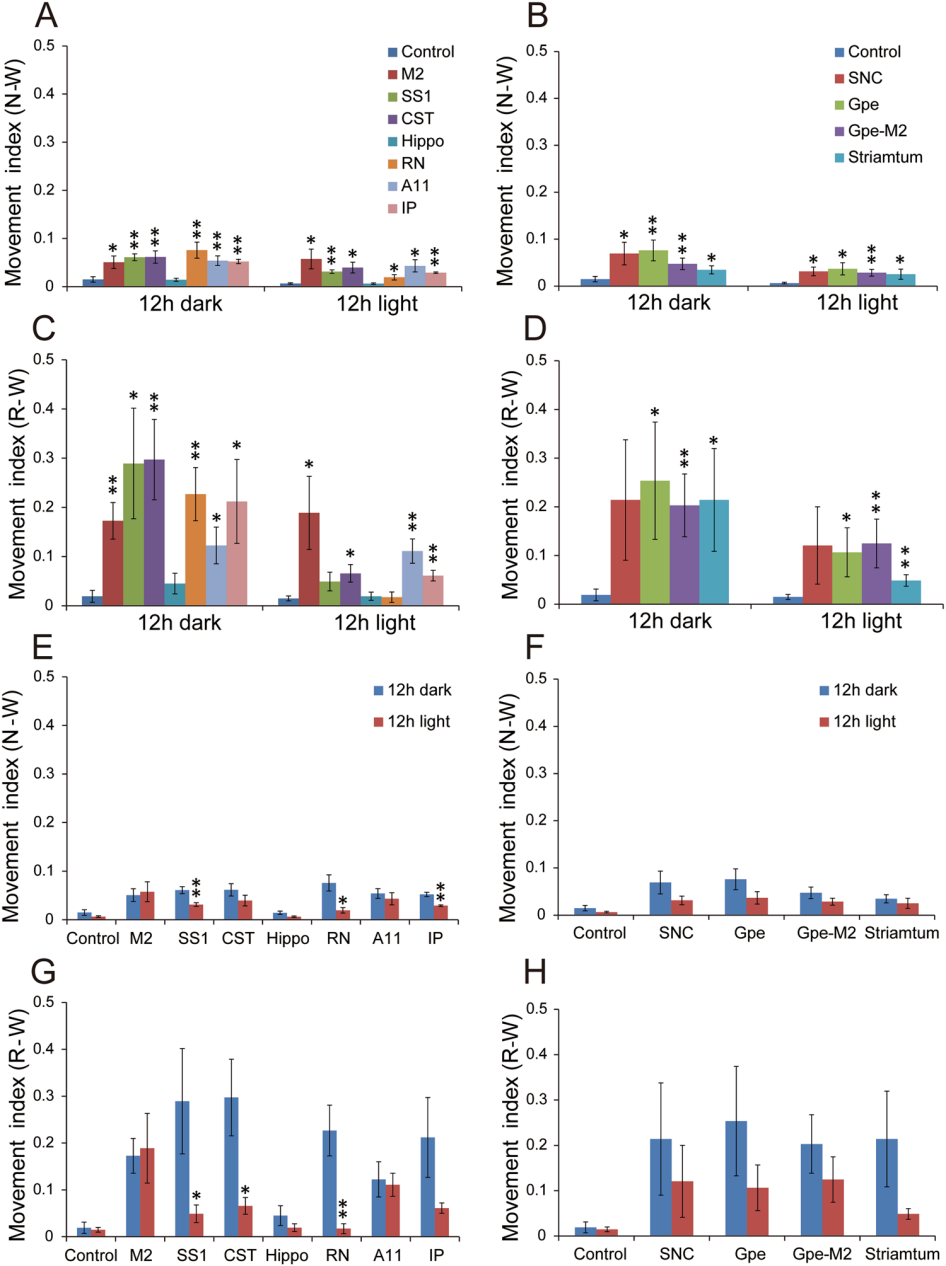
Movement index (MI) of damages to BG-corticospinal, cerebello-rubro-spinal systems, and A11. As a majority of RLS-like movements occur during sleep-wake transitions, we introduced and calculated a movement index (MI = RLS-like movement amounts per sleep-wake transition) for quantification of the severity of RLS-like movements. MI changes are mostly in line with total amounts of RLS-like movements. *p < 0.05, **p < 0.01.

**Figure 6:**
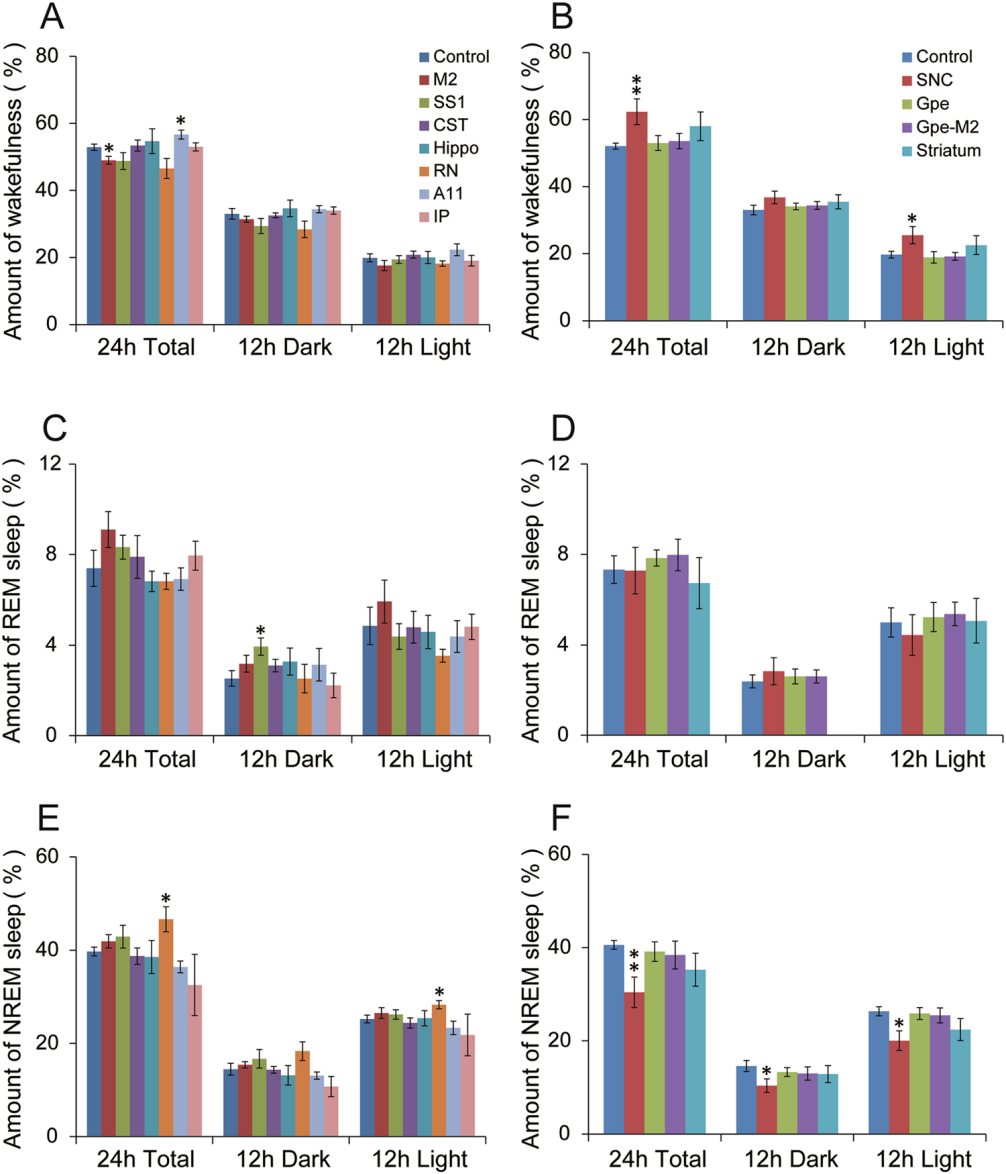
Daily sleep-wake amounts of damages to BG-cortico-spinal, cerebello-rubro-spinal systems, and A11. Of all lesion groups, only unilateral SNc lesions and bilateral RN lesions decreased and increased total NREM sleep amounts (24 hours) significantly. *p < 0.05, **p < 0.01.

**Figure 7:**
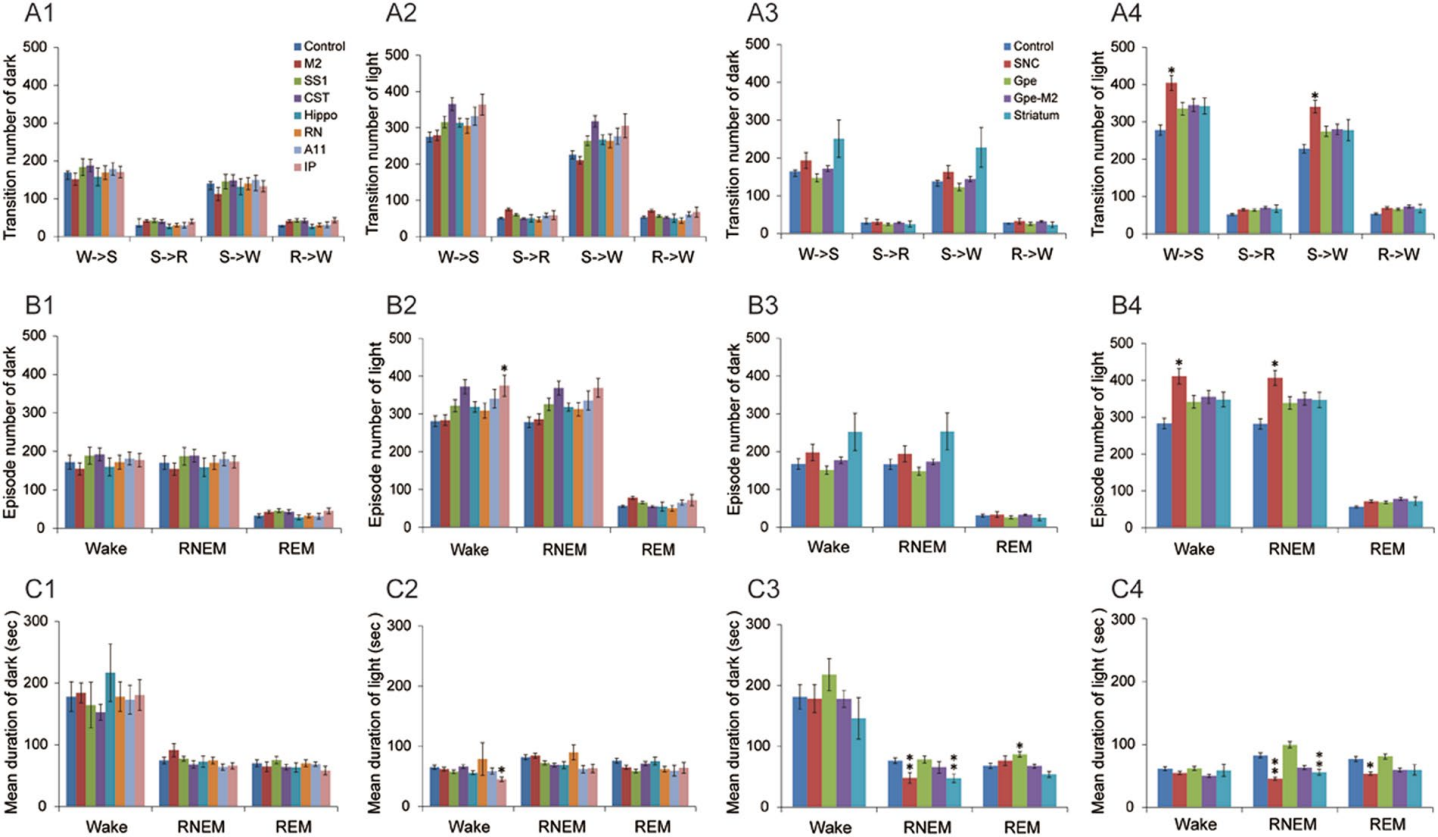
Sleep-wake state stability of damages to BG-cortico-spinal, cerebello-rubro-spinal systems, and A11. Of all lesion groups, unilateral SNc lesions caused significant sleep-wake fragmentations (changes in bout duration and bout number). *p < 0.05, **p < 0.01.

Compared to controls, rats with CST lesions exhibited significantly more RLS-like movements (Figs 2 and 3), with many of the RLS-like movements observed during N-W (p = 0.004/L, 0.007/D, 0.001/L + D) and R-W (p = 0.004/L, 0.002/D, 0.0001/L + D) transitions (Figs 3 and 4). RLS-like movements were not seen during NREM sleep. Although RLS-like movements were higher during REM sleep, the difference did not reach significance. RLS-like movements were not seen in wake transitions to sleep. Overall, RLS-like movements were more vigorous during the night than during daytime. The probability of RLS-like movements per transition (RLS-like movements amounts/N-W and R-W transition times) was higher during nighttime than during daytime (Fig. 5). We did not observe significant changes in sleep-wake amounts or patterns following CST lesion (Figs 6 and 7), compared to control rats.

The motor cortex is very large, and so for this study we targeted the most frontal level, the secondary motor cortex (M2), as it is strongly targeted by the anterior external globus pallidus (GPe) of the basal ganglia. At this particular level (Fig. 1), only M2 exists (M1 remains situated caudally), where M2 solely contributes to the CST. We also chose to target the somatosensory cortex (SS1) as it is far enough from M2 to prevent lesion overlap.

Because M2 and SS1 span a considerable portion of the rostrocaudal axis, we never achieved completely ablation of these cortical areas. Instead all of our lesions were partial. Similar to CST lesion, both M2 and SSI lesions significantly increased RLS-like movements in sleep-wake transitions (N-W and R-W) (M2: N-W, p = 0.073/L, 0.035/D, 0.04/L + D; R-W, p = 0.082/L, 0.007/D, 0.031/L + D) (SS1: N-W, p = 0.002/L, 0.001/D, 0.00001/L + D; R-W, p = 0.128/L, 0.009/D, 0.003/L + D). Unlike CST damage, M2 and SS1 lesions increased RLS-like movements during NREM sleep and REM sleep (M2 N: p = 0.01/D, 0.07/D, 0.003/L + D; R: p = 0.19/L, 0.023/D, 0.018/L + D) (SS1 N, p = 0.01/L, 0.007/D, 0.003/L + D, R, p = 0.196/L, 0.023/D, 0.018/L + D), although total amounts of abnormal movements during NREM sleep and REM sleep were much lower than RLS-like movements during sleep-wake transitions (Figs 3, 4 and 5). RLS-like movements in general were more vigorous during the night than during the daytime. The probability of RLS-like movements per transition was higher during night than during daytime (Figs 4 and 5).

As an anatomical control, we made lesions within the hippocampus in 5 rats (Fig. 1). We chose the hippocampus on the basis that this structure has no descending projections to the brainstem or spinal cord. Hippocampal lesions in our study were mostly confined to the dorsal hippocampus and clearly eliminated theta EEG during REM sleep, but showed no significant effects on sleep-wake amounts including REM sleep or circadian pattern, and had similar minimal movements during sleep and sleep-wake transition as controls (Figs. 4–7).

These results indicate that CST and its cortical sources (M2 and SS1) regulate RLS-like movements during sleep-wake transitions. Cortex (M2 and SS1) also regulates RLS-like movements during NREM sleep and REM sleep, via extra-CST.

#### Role of cerebello-rubro-spinal cord network in RLS-like movements

The rubrospinal projection together with the corticospinal projection forms the two primary descending glutamatergic inputs for general motor control. The red nucleus (RN) also receives inputs from the motor cortex, somatosensory cortex, and cerebellar deep nuclei—especially the cerebellar interposed nucleus (IP). To examine the role of the cerebello-rubro-spinal network in RLS, we placed lesions in the RN and IP.

Bilateral RN lesions (N = 5) generated using ibotenic acid and confirmed by Nissl staining (Fig. 1) resulted in a significant increase in RLS-like movements during N-W (p = 0.054/L, 0.001/D, 0.0001/L + D) and R-W (p = 0.64/L, 0.004/D, 0.012/L + D) transitions as well as during NREM sleep during dark and 24 hour period (p = 1.0/L, 0.035/D, 0.035/L + D). A non-significant trend towards an increase in RLS-like movements during REM sleep was also observed (Figs 3, 4 and 5).

Bilateral cerebellar IP lesions (N = 5) were generated using ibotenic acid and verified by NeuN and Nissl staining (Fig. 1) produced similar changes as RN lesions, RLS-like movements significantly increased during N-W (p = 0.0001/L, 0.003/D, 0.0001/L + D) and R-W (p = 0.02/L, 0.007/D, 0.005/L + D) transitions. During NREM sleep and REM sleep, significant increase was only seen during NREM sleep (p = 0.035/L) (Figs 3, 4 and 5).

RN lesions also showed a significant increase in NREM sleep amounts (Fig. 4). Sleep-wake duration and bout analysis showed no significant alteration by RN and IP lesions (Figs 6 and 7).

These results indicate that rubrospinal projection regulate RLS-like movements during sleep and transitions. Specifically, M2 and SS1 may regulate RLS-like movements during sleep via RN, whereas the cerebellum may do so via the IP-RN pathway.

#### Role of dopamine descending projections in RLS-like movements

The hypothalamic A11 dopamine group is the only dopaminergic group that sends projections to the spinal cord, and this cell group has long been implicated in RLS and periodic leg movements (PLM). The A11 group is located in the caudal hypothalamus, dorsal and lateral to the third ventricle, and also provides innervation of the cerebral cortex.

To selectively lesion A11 descending projections, we injected 6-OHDA into the dorsolateral region of the C1 cord, where A11 descending axons pass through, in 6 rats. Two weeks after injections we recorded EEG/EMG/video for 24 hours. We also administer the dopamine D2/D3 agonist pramipexiole, a first-line medication for treating RLS and PLM in humans, to the A11-lesioned rats to ascertain effects on RLS-like movements during night. Finally, the rats were perfused and brains were histologically processed.

Because A11 neurons projecting to the spinal cord locate at the caudal level, we selected the caudal A11 to count loss of dopaminergic neurons. When we quantified loss of TH + cells in the A11 region, we found that 6-OHDA produces only ~40% reduction in the number TH + cells in the caudal A11, as compared to controls (Fig. 1). Given that A11 neurons also project to other sites such as the cortex, it is possible that TH + A11 cells loss was specific to those projecting to the spinal cord. The TH-ir neurons in A5–7 groups were not affected (not shown). Because TH terminals in the spinal cord are also from the A5–7, using TH label to determine loss of A11 projections may not provide a reliable quantification of cell loss.

Despite incomplete loss of A11 cells, we observed a significant increase in RLS-like movements during N-W (p = 0.039/L, 0.005/D, 0.003/L + D) and R-W (p = 0.005/L, 0.044/D, 0.001/L + D) transitions but not during NREM sleep and REM sleep (Figs 3, 4 and 5).

There were no changes in total amounts of sleep-wake in the A11 lesioned group although a reduction in the duration of REM sleep bouts was observed (Figs 6 and 7).

#### Pramipexole reduces RLS-like movements and promotes NREM sleep

In A11 lesioned group, we also administered 0.5 mg/kg of the D2/D3 agonist pramipexole (or saline) at 6 PM and recorded EEG/EMG/video for overnight. We compared the number of RLS-like movements between saline and pramipexole treatment during the 19:00–7:00 time window.

**Figure 8:**
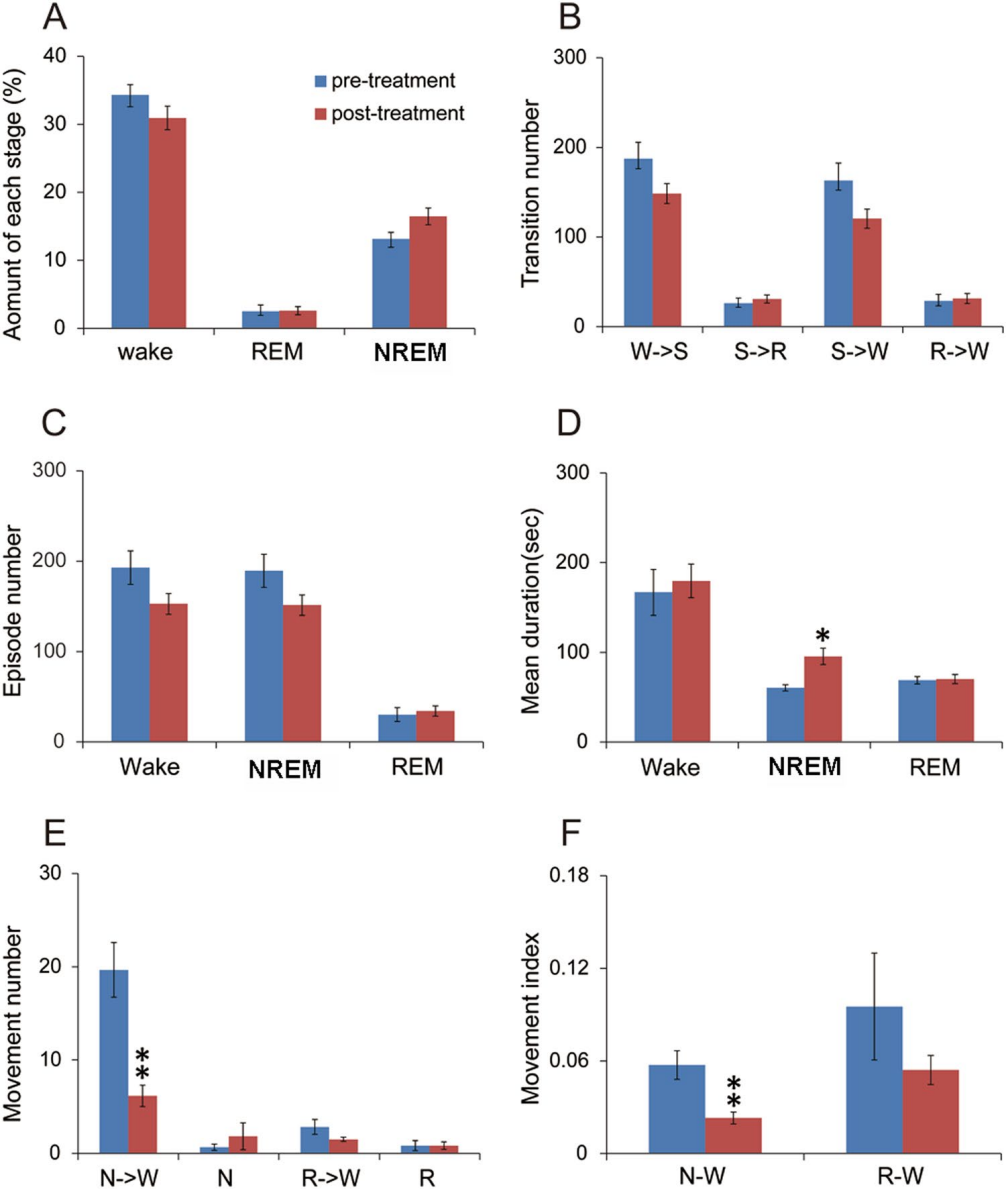
Pramipeixole reduces RLS-like movements and increases NREM sleep. Pramipeixole (0.5 mg/kg) injected (ip) at 6 PM significantly reduced RLS-like movements and MI in N-W transitions during the night in rats with A11 lesions, compared to that of saline injection in the same rats at 6 pm of the prior day. Pramipeixole also significantly increased NREM sleep, by lengthening the bout durations. *p < 0.05, **p < 0.01.

We found that pramipexiole significantly reduced RLS-like movements in the A11 lesioned group during N-W transitions, compared to saline injection. Furthermore, pramipexole increased NREM sleep amounts (Fig. 8), which linked to a specific increase in average bout duration (Fig. 8). However, because we only injected the drug at one-time point and at one dose, more studies are needed to fully characterize the efficacy of pramepexiole in treating RLS-like movements and on sleep at different times as well as to test efficacy in the RN and other lesioned groups. Nevertheless, reduction of RLS-like movements by pramipexole suggests, albeit indirectly, that rodent RLS-like movements resemble human RLS.

### Role of BG in RLS-like movements

#### Unilateral SNc lesion (striatal dopamine depletion)

The BG and cortex have a complex interrelationship, with disturbances in BG altering cortical activity, including motor-sensory cortical activity, which via the corticospinal pathway may induce RLS-like movements. Our prior series of studies on BG have suggested that nigrostriatal dopamine, via presynaptic D2 receptors at striatopallidal axons, may activate the GPe, whereas pallidocortical neurons likely regulate cortical activity to influence sleep, motor activity and, possibly, RLS.

To investigate the role of dopamine in the BG but also avoid the potential confound of changes in food intake and body weight (as previously documented in animals with bilateral SNc lesions), we placed unilateral injections of 6-OHDA into the ventral region of the globus pallidus (GPe) in 5 rats, as previously described^13, 21^. After two weeks, we recorded EEG/EMG/video for 24 hours. For control, we injected saline into the third ventricle in 6 rats and recorded EEG/EMG/video for 24 hours. This control was also used to compare to animals with lesions in the GPe, striatum, and pallidocortical neurons.

**Figure 9:**
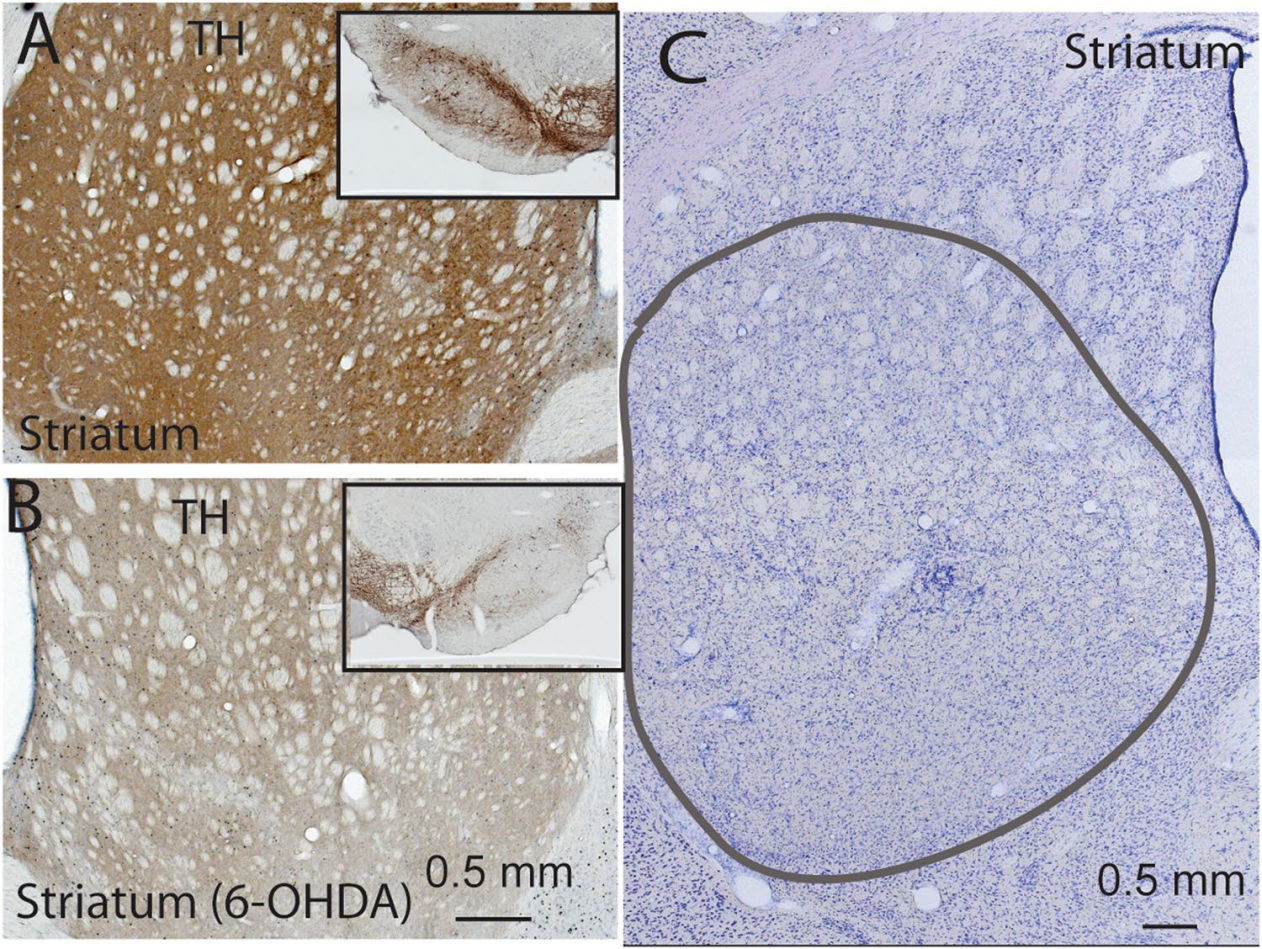
Histology of SNc and striatal lesions. Loss of dopaminergic SNc (insert box) and its efferents in the striatum are shown in B, compared to intact SNc (**A**). Striatal lesions are made by ibotenic acid (**C**). Because of the large size of the striatum, our lesions were focused on the lateral striatum, which is involved in motor regulation.

Compared with the intact (non-lesion) side, dopaminergic projections to the dorsal striatum but not ventral striatum (or nucleus of accumbens) were eliminated on the lesioned side (Fig. 9). Consistent with this, SNc but not VTA, dopaminergic neurons were killed (Fig. 9). Compared with controls, the number of RLS-like movements in 12 hours light period, 12 hours dark period and 24 hours period was significantly higher in N-W (p = 0.021/L, 0.003/D, 0.004/L + D) and R-W (p = 0.037/L, 0.062/D, 0.002/L + D) transitions, but not during NREM sleep or REM sleep (Figs 3, 4 and 5).

A significant reduction in NREM sleep (wake increase) was observed during the daytime (p = 0.047) and 24 hours period (p = 0.004) (Fig. 6), although the wake itself was fragmented (frequent wake transitions and short wake duration) (Fig. 7). The magnitude of sleep changes induced by unilateral SNc lesion was far less than that observed following bilateral SNc lesion in our previous work^13^.

#### Unilateral striatal lesions

As SNc dopamine targets the striatum, we sought to explore the role of this afferent input in the development of RLS-like movements. To maintain consistency with our SNc lesions, which were unilateral, we made unilateral striatal lesions by injecting ibotenic acid in the striatum in 5 rats.

Histological analysis revealed that all lesions were confined to the dorsolateral striatum (Fig. 9). Unilateral striatal lesions produced increase in RLS-like movements in transitions in N-W (p = 0.012/D, 0.058/L, 0.012/L + D) and R-W (p = 0.001/L, 0.09/D, 0.005/L + D) (Figs 4 and 5). Unilateral striatal lesions did not increase RLS-like movements during NREM sleep and REM sleep.

Unlike bilateral striatal lesions^22^, unilateral striatal lesions did not affect sleep-wake amounts or sleep-wake transitions (Figs 6 and 7).

#### Unilateral pallidal (GPe) lesions

**Figure 10:**
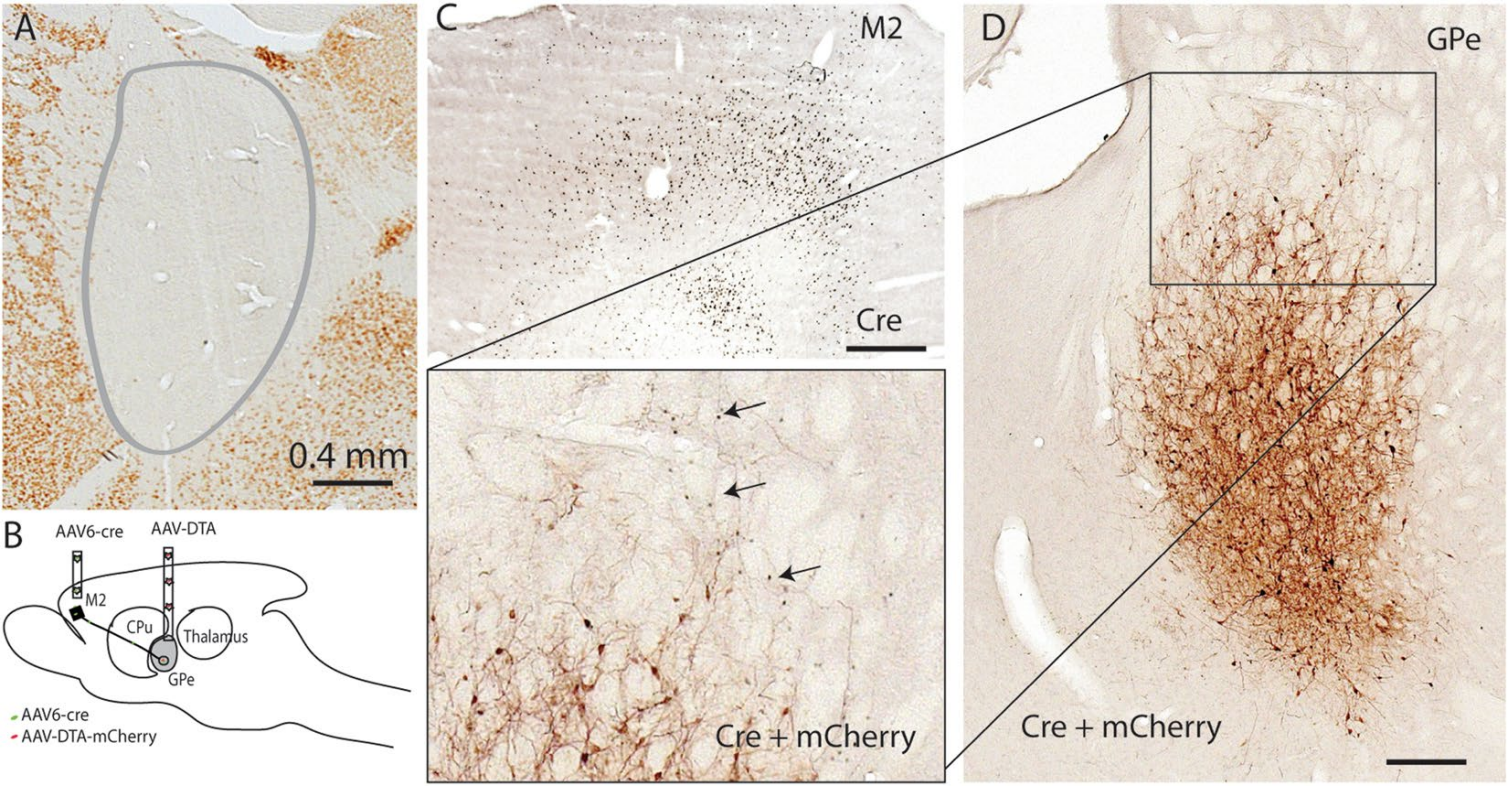
Histology of lesions in GPe and pallidocortical neurons. GPe lesions are made by ibotenic acid (**A**). Selective lesions of pallidocortical neurons are produced by AAV6-cre injections in M2 and cre-dependent AAV10-DTA into the GPe (**B**–**D**). Because we only inject AAV6-cre into M2 (**C**), cell loss in the GPe is confined to pallidocortical neurons projecting to the M2 (**D**), pallidocortical neurons projecting to other cortical regions are not affected. Arrows point cre labeled neurons that are not exposed to AAV-DTA-mCherry while cre is not seen in presence of AAV-DTA-mCherry (brown color), indicating indirectly that cre labeled pallidocortical neurons are killed.

Again, to avoid the loss of body weight associated with bilateral GPe lesions, we made unilateral GPe lesions using ibotenic acid in 5 rats (Fig. 10). After two weeks, we recorded EEG/EMG/video for 24 hours.

Similar to unilateral SNc lesions, unilateral GPe lesions significantly increased RLS-like movements in N-W and R-W, but not during NREM sleep or REM sleep in 24 hours period. During the 12 hour light period, movements were significantly higher in N-W (p = 0.017) and R-W (p = 0.024) transitions as well as during REM sleep (p = 0.002), while movements were not significantly affected during R-W transitions and during NREM sleep. During the dark period, movements were significantly higher than control in N-W (p = 0.012) transitions while the movements during NREM sleep and REM sleep were not significantly different from control (Figs 3, 4 and 5).

Sleep-wake stage and amounts analysis showed no alteration in sleep amounts and pattern by unilateral GPe lesions, compared to control (Figs 6 and 7). Bilateral pallidal lesions reduce sleep by almost 50%^22^.

#### Selective lesions of pallidocortical neurons

Pallidocortical neurons in the GPe topographically project to the cerebral cortex^17, 18^. To selective target and ablate these projection neuron, we placed bilateral injections of a retrograde AAV expressing Cre-recombinase into M2, and a cre-recombinase dependent cell toxin (AAV10-DTA) into the GPe, also bilaterally, in 5 rats. After two weeks, we recorded EEG/EMG/Video for 24 hours.

Loss of pallidocortical neurons was indirectly confirmed by absence of cre in mCherry field and presence of cre in GPe that was not filled by mCherry (AAV10-DTA). The mCherry-labeled neurons were pallidal neurons without cre (Fig. 10).

**Figure 11:**
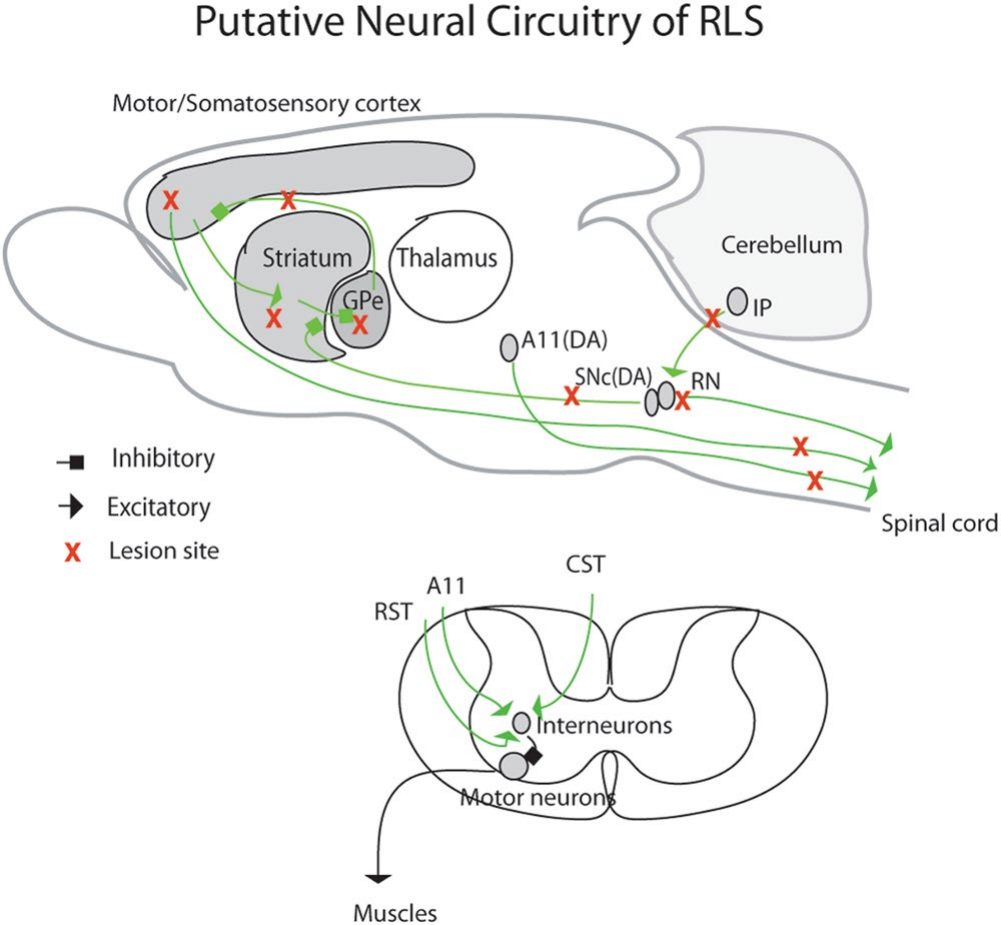
Putative neural circuits of RLS. Corticospinal tract (glutamate), rubrospinal tract (glutamate) and A11 (dopamine) projections converge onto glycine/GABA interneurons, inhibiting motor activity during sleep and sleep-wake transitions. The BG, via the cortex, and the cerebellum, via the red nucleus, modulate motor activity. Disruptions of these circuit nodes marked by red color cross result in RLS.

Compared to controls, significantly more RLS-like movements were observed during N-W (p = 0.017/L, 0.012/D, 0.001/L + D) and R-W (p = 0.024/L, 0.054/D, 0.001/L + D) transitions, and more RLS movements were seen during REM sleep (p = 0.02/L, 0.19/D, 0.026/L + D) (Figs 3, 4 and 5). We did not find significant changes in sleep-wake amounts and patterns and sleep-wake transitions (Figs 6 and 7). Similar results for the RLS-like movements following lesions in the GPe, GPe-M2 and M2 suggest that regulation of RLS-like movements pallidocortical pathway links to BG control of the cortex (Fig. 11).

## Discussion

Our results reveal, for the first time, that three supra-spinal inputs to the spinal cord, i.e., corticospinal tract (CST), rubrospinal tract and A11 dopaminergic cell population, all contribute to the suppression of motor activity and movements during both sleep and transitions into quiet wakefulness from sleep (N-W and R-W). Targeted lesions of these pathways and their afferent sources (SS1, M2 and cerebellar IP) induced RLS-like movements, with the most severe and vigorous movements consistently occurring during the night period. Administration of the dopamine D2 agonist pramipexole also reduced RLS-like movements in the A11 lesioned group, which was the only lesion group to receive pramipexole. Finally, we show that pallidocortical neurons of the BG exert important control over the motor cortex during sleep. A summary of these putative neural pathways regulating RLS is outlined in Fig. 11.

One of the primary clinical features of RLS in humans is akathesia, which is characterized as an inner impulse to move all or parts of the body, the limbs in particular. These sensations are most commonly felt during quiet wake and during transitions from wake into sleep. As compared with humans, rodents have more frequent sleep-wake transitions, shorter wake-sleep episode durations, and sleep episodes that are distributed across the day and night. Yet despite this difference in sleep architecture, RLS-like moments in our rat models were particular prominent during sleep-wake transitions and quite wakefulness, mirroring that of humans with RLS. Thus, this feature of rodent RLS along with the clinically positive response to pramipexole suggests that our rat model of RLS recapitulates several key features of human RLS. One notable difference is that RLS-like movements in our rat models were rarely seen in wake transitions into sleep. One prominent feature of human RLS is that symptoms intensify in late day and earlier night. Consistent with this temporal feature, we found that RLS-like movements in our rats models were vigorous and violent during that the second half of night. Thus while more evidence is needed to confirm that RLS-like movements in our rat model are equivalent to those of human RLS, our study does clearly delineate multiple descending pathways that regulate motor activity during sleep.

Due to the large sizes of our targeted regions, e.g., M2, SS1 and hippocampus, some of our lesions were only partial. In cases of the BG, the lesions were unilateral. In other cases, such as the CST, IP, and RN, our lesions were nearly complete. Thus, we have avoided making direct comparisons of the amount of RLS-like movements and sleep-wake time between lesion groups. It is of interest that unlike bilateral lesions of the BG that strongly alter sleep-wake behavior^13, 22^, unilateral BG lesions have either small or no effects on sleep-wake architecture but did produce RLS-like movements, highlighting a particularly strong link between the BG and motor control during sleep. The potential role of the RN in wake promotion is intriguing but will require further investigation.

Normal rats may have very few RLS-like movements during REM sleep (Type I), whereas rats with lesions of the CST, rubrospinal pathways (source or projections) or A11 dopamine cell group all exhibited pronounced RLS-like movements (type I and II). One important feature of these RLS-like movements was their stereotypic nature, which took the form of 2–5 sequential movements in a 10 s or less time interval (i.e., Type II), and was never observed in control rats. We also failed to observe these RLS-like movements in rats with lesions of the hippocampus. This is an important point given that 1): the hippocampus has no direct link to the CST, rubrospinal system or A11 cell groups and 2): rules out a non-specific effect of the lesions.

As indicated, and similar to human RLS, most of the RLS-like movements we observed in the lesioned rats occurred during sleep-wake transitions and quiet wakefulness. Interestingly, and in comparison to other lesion groups, lesions of the M2 or SS1 cortex or the RN resulted in an increase in RLS-like movements during NREM sleep, suggesting that the cortex may rely in part on the cortico-rubro-spinal pathway for suppression of motor activity during NREM sleep. Lesion of M2 or SS1 or GPe or pallidocortical neurons significantly increased phasic movements (Type I) during REM sleep. We hypothesize that cortical projections to the ventromedial medullar (VMM) may be critically involved in suppressing motor activity during REM sleep. Within the pontine-medullary brainstem, two spinally-projecting cell populations have been identified as playing a key role in mediating the atonia of REM sleep. The glutamatergic sublaterodorsal tegmental nucleus (SLD), located in the dorsolateral pons, projects to and activates glycine-GABA interneurons of the spinal ventral horn to suppress muscle tone during REM sleep. The VMM, which also receives inputs from the SLD, but instead uses glutamate and glycine/GABA to regulate atonia during REM sleep^8^. And while lesions of the SLD or VMM produce REM without atonia, they do not alter motor suppression during NREM sleep or quiet wakefulness^3, 9^. Because both motor and somotasensory cortices project to the VMM and cortical lesions increase RLS-like movements during REM sleep, we speculate that cortico-medullo-spinal pathway may be critically involved in suppression of movements during REM sleep. We also previously showed that disruption of another spinally-projecting group of brainstem neurons, located with the mesencephalic locomotor region (MLR), induces cataplexy and freezing behaviors, but does not affect motor suppression during sleep and sleep-wake transition^3, 10^.

While it is relatively common for RLS patients to express periodic leg movements (PLM), most patients with PLM do not have RLS^23^. Thus, RLS is probably a severe and extreme subtype of PLM. PLM occurs during sleep, in particular during NREM sleep, and are not typically accompanied by changes in the EEG. If we assume that RLS-like movements observed during NREM and REM sleep are PLM-like movements, then the number of PLM-like movements during NREM and REM sleep in lesion groups was actually relatively low (<10 per 24 hours), and it appears that only cortical, pallidocortical and RN lesions significantly increase PLM movements during NREM and REM sleep. There is one literature report of PLM like movements in rats with A11 lesions^24^, which we did not observe in our A11 group lesion group. Our data suggest that rats may exhibit a weak PLM phenotype, and that the cortico-rubro-spinal pathway is involved.

While our data clearly indicate that all three of the supraspinal systems under study contribute to the suppression of motor activity during sleep and wake-sleep transitions, it remains to be clarified how motor suppression is achieved at the level of the spinal ventral horn. We hypothesize, but have not demonstrated, that this is achieved through modulation of GABA/glycine interneurons within the spinal ventral horn. These inhibitory interneurons receive glutamatergic and dopaminergic inputs from the descending corticospinal (glutamate), rubrospinal (glutamate) and A11 (dopamine) systems (Fig. 11). This represents an important area of future investigation.

A role for the BG in the regulation of motor behavior and sleep is well established. Given that BG strongly regulate motor activity during wake, vis-a-vis modulation of the cerebral cortex, it is possible, if not likely, that the BG continue to influence motor activity, or suppression thereof, during sleep. Recent lesion studies in different species have suggested a particularly important role for the GPe in motor and sleep regulation as well as Parkinson’s disease^25^, and that this regulation may reflect direct pallidal regulation of the cortex that is independent of thalamic activity. To this end, three recent studies, including two studies from our group, has revealed that the GPe has direct cortical projections^17–19^. Furthermore, thalamic lesions are without affect on total sleep amount^26^ and they fail to induce RLS-like movements (unpublished observations). Results from the present study provide support for the concept that pallidocortical neurons innervating M2 may suppress motor activity during sleep and sleep-wake transitions. Of note, and in contrast to non-selective bilateral GPe lesions that are severely sleep disrupting, lesions that are restricted to the pallidocortical neurons that innervate M2 do not significantly alter sleep, but rather selectively disrupt motor suppression during NREM sleep. Sleep control may therefore link to a specific subset of pallidocortical neurons that target extensive cortical regions.

In rats and cats, Lai and colleagues have shown that non-selective lesions of the retrorubral field (RRF) or infusion of a GABA agonist into the inferior colliculus produces PLM^27, 28^. Yet close inspection of the movements shown in these papers indicate that the movements are associated with a waking EEG, suggesting that these movements are similar to what we have observed. And while Lai and colleagues propose that RRF GABAergic neurons - via medullary reticulopsinal neurons - are involved in PLM, our data indicate that loss of dopamine neurons within the SNc and RRF subserve the appearance of the abnormal movements.

In humans, the genes BTBD9, MAP2K5, MEIS1 have been recently associated with human RLS^29^. The products of these three genes are highly expressed in the BG (BTBD9, MAP2K5, and MEIS1), cerebral cortex (BTBD9), RN (MAP2K5) and spinal cord (BTBD9, MAP2K5, and MEIS1), all of which are components of the proposed RLS circuitry identified in our study (Allen Brain Atlas). Iron deficiency is also strongly associated with RLS and so it is of interest that low levels of iron have been detected in the BG and RN^30–33^. Moreover, strokes occurring within the BG, internal capsule, pons and cerebellum are strongly associated with the development of RLS^34, 35^. Finally, transcranial magnetic stimulation (TMS) over motor cortex reduces RLS^36, 37^. Thus it is likely that the anatomic, genetic and biochemical etiological bases of RLS is diverse. Data from the present study provides a circuitry blueprint for further investigating and identifying specific causes, treatments and neural mechanisms of RLS.

## Methods and Materials

### Animals

Animal protocols were approved by Beth Israel Deaconess Medical Center Animal Care and Use committees and in accordance with the guidelines with institutional animal care and use committees (IACUC). Male Sprague Dawley rats (275–300 gm; Harlan Sprague Dawley, Indianapolis, IN) were housed under controlled conditions (12:12 light-dark cycle, light on = 07:00 AM, 100 lux) in an isolated ventilated chamber maintained at 20–22 °C, with food and water available *ad libitum*.

### Lesion surgeries

Animals were anesthetized by ketamine and xylazine. For ibotenic acid lesions, 10% ibotenic acid (30–60 nl) was injected into the intended areas: M2 (AP, 3.5 mm, ML = 2.0 mm, DV = 2.0 mm), SS1 (AP, −1.0 mm, DV, −2.0 mm, ML, 5.0 mm), hippocampus (AP, −4 mm, DV, 2.8 mm, ML, 2.5 mm), RN (AP, −6 mm, DV, −6.8 mm, ML, 0.8 mm), IP (AP, −11.6 mm, DV, −6.6 mm, ML, 0.8 mm), striatum (AP, 0 mm, ML, 3.0 mm, DV, 5.0 mm), GPe (AP, −0.5, ML, 3.0 mm, DV, 6.0 mm).

For CST lesion, we placed a coronal cut in the dorsal region, at the C1 level, where corticospinal projection pass through. For A11 lesions, we injected 60 nl of 6% 6-OHDA into the dorsolateral region at C1 level where A11 dopaminergic projections passing through bilaterally. For SNc lesions, 50 nl 6% of 6-hydroxydopamine hydrochloride (6-OHDA, H4381, sigma) in saline solution was injected into the SNc using predetermined coordinates (AP = −0.5 mm, ML = 2.4 mm, DV = 7.5 mm). For lesions of pallidocortical neurons, bilateral injections of a retrogradely transported cre-recombinase viral vector (AAV6-cre; 300 nl, Boston Children Hospital Core) and a cre-dependent cell toxin, 120 nl Diptheria Subunit A (lox-mCherry-lox-DTA-WPRE-AAV, serotype 10; DTA-AAV produced and validated by P. Fuller and M. Lazarus) were placed into M2 cortex and the GPe, respectively. Hence, cre recombinase was retrogradely transported from M2 to the GPe, where it triggered expression of the Diptheria Subunit A toxin, resulting in selective ablation of pallidocortical neurons that project to M2. Control animals received the same surgery as the lesion group with the exception that they received saline injections into the third ventricle. It could be argued that saline injections into the same targets as the toxin injections would be a better control. This, however, would have required substantially more animals. In our experience, those controls should not differ from the control we employed. Because these experiments took a long time (2 years), we had two control groups. One control was used to compare to the lesion in corticospinal pathway (CST, M2, SS1, hippocampus), cerebello-rubro-spinal pathway (RN and IP) and dopamine inputs to the spinal cord (A11) and the other control group was used to compare to the lesion in BG (SNc, GPe, striatum, GPe-M2).

List of numbers of animals in each group: N = 8 (control), N = 6 (M2), N = 6 (SS1), N = 5 (CST), N = 5 (Hippocampus), N = 5 (RN), N = 6 (A11), N = 5 (IP); N = 6 (control), N = 5 (SNc), N = 5 (GPe), N = 5 (Striatum), N = 5 (GPe-M2). Lesion in SNc, GPe and striatum was unilateral and all other lesion groups were bilateral. Due to miss injections and EEG/EMG caps coming off, some rats were excluded.

### Histology

All rats (of lesions and controls) were deeply anesthetized by chloral hydrate (500 mg/kg), perfused with 0.9% saline followed by 10% neutral buffered formalin (Sigma) through the heart. The brains were sectioned on a freezing microtome at 40μm into 4 series. We used diaminobenzidine (DAB) reaction to label tyrosine hydroxylase (TH), mCherry, neuN and cre.

Briefly, tissues were incubated with TH (1:20 K, #22941, Dasorin), mCherry (1:5 K, #632496, Clontech), neuN (1:1 K, Chemicon) and cre (1:1 K, #69050, Novagen) for 24 hours. The tissues were then incubated in biotinylated anti-primary antibodies for one hour and then avidin/biotin mixture solution for one hour and finally exposed to DAB in presence of hydrogen peroxide. Floated sections were then mounted on slides, dried and dehydrated and covered.

To determine loss of A11 neurons to the spinal cord, TH-labeled neurons were counted at level (AP = −3.8 mm) in two adjacent sections with 120 mm space. At this level, A11 DA neurons project to the spinal cord.

### Sleep-wake and RLS-like movement analysis

EEG, EMG and video were recorded (256 Hz sampling) and analyzed by SleepSign System (Kissei Inc, Nagnao, Japan). We observed two classes of movements during sleep and sleep-wake transitions in lesioned animals. Type I movements were characterized by rapid jerking of the limbs, head or entire body and single or sequential EEG spikes (Fig. 1), with a single narrow large EEG spike during the NREM to wake (N- > W) or REM to wake or during quiet wakefulness (R- > W) transitions. Type II movements were characterized by body or limb twitching and myoclonus with duration of 1–2 seconds (Fig. 2). These Type II movements typically expressed as 2–5 sequences within a 1–5 seconds intervals. In normal control rats, Type 1 movements were occasionally observed only during REM sleep ( < 3 per 24 hours). Type II movements were almost never observed in unlesioned controls, but were frequently observed in rats with lesions within the three supra spinal systems. RLS-like movements were hence defined on the basis of both Type I and Type II movements occurring in the same animals. Most RLS-like movements occurred at transition into quiet wake wakefulness during which rats remained sleep posture (Figs 1 and 2).

In more detail, Type I movements were identified by a unique spike in the EMG and confirmed through analysis of time-locked video. Video confirmation was necessary as occasionally a big spike was observed in the EMG but only produced relatively small jerking (likely due to the placement of the EMG electrode in the nuchal muscles). Type II movements could only be ascertained through analysis of video. In the lesioned animals, Type I and II movements were mostly observed during sleep transitions into wakefulness (N-W and R-W transitions) and subsequent quiet wake. We counted RLS-like movements occurring precisely during N-W transitions or during the following wake episode as # in N-W transition. Similarly we counted RLS-like movements in R-W transitions. We counted RLS-like movements observed only during NREM sleep and REM sleep as # in N and # in R. Small movements, even those with large EMG spikes, were not counted, whereas big movements, including those with small EMG changes, were counted.

For consistence in identifying abnormal movements and avoiding bias, one researcher (WJY) did all sleep and movement analyses for three times.

### Pharmacological treatment

Pramipexole solution (2.0 mg/ml) was prepared by 10 mg pramipexole (Santa Cruz Biotechology, Lot#B1412) in 5 ml saline. We administered saline at 18:00 and 0.5 mg/kg pramipexole at 18:00 at the following day, and recorded EEG/EMG/video overnight for both days. Analyses were done for sleep-wake behavior and RLS-like movements for two nights. Although we did not examine the effects of other doses of pramipexole on sleep and RLS-like movements, 0.1–1.0 mg/kg range of pramipexole is widely used for rat behavioral research^38, 39^.

### Statistical method

Because difference in lesion sizes ranging from partial, unilateral, and bilateral lesions, it is not proper to compare between lesion groups. We are interested in finding the difference between each lesion group vs control. Thus Student T-test was used to determine significance differences (p < 0.05, p < 0.01) between lesion and control group. We first determined that the data were normally distributed, and then T-test and p-value were generated by SPSS statistics 21.0.

## Electronic supplementary material

Videos of normal and RLS-like movements in rats

Normal movements during sleep-wake transitions

RLS-like movements during sleep-wake transitions
