# Supplementary material for: Targeted disruption of supraspinal motor circuitry reveals a distributed network underlying Restless Legs Syndrome (RLS)-like movements in the rat
Source: Sci Rep. 2017 Aug 29;7:9905. doi: 10.1038/s41598-017-10284-3 (PMC5575019; doi:10.1038/s41598-017-10284-3)
Supplement: Supplementary file 1 — Videos of normal and RLS-like movements in rats [file 41598_2017_10284_MOESM1_ESM.pdf]

Targeted disruption of supraspinal motor circuitry reveals a distributed network underlying Restless Legs Syndrome (RLS)-like movements in the rat

Chun-Ni Guo[1,5#'] , Wen-Jia Yang[2,5#], Shi-Qin Zhan[3,5], Xi-Fei Yang[4,5], Michael C. Chen[5], Patrick M. Fuller[5], Jun Lu[5*]

[1]Department of Neurology, Shanghai First People's Hospital Affiliated to Shanghai Jiaotong University, Shanghai, China
[2]Shanghai Yueyang Integrated Medicine Hospital, Shanghai, China
[3]Department of Neurology, Xiuwu Hospital, Capital Medical University, Beijing, China
[4]Shenzen Centers for Disease Control and Prevention, Guangdong, China
[5]Department of Neurology and Division of Sleep Medicine, Beth Israel Deaconess Medical Center and Harvard Medical School, CLS 709, 3 Blackfan Circle, Boston, MA 02115

#CNG and WJY contribute equally
*Corresponding author: jlu@bidmc.harvard.edu

Video 1 shows normal movements during sleep-wake transition in a control rat while video 2 shows RLS-like movements during sleep-wake transitions in a rat with bilateral lesions of the red nucleus.
